# Development and validation of ultra performance liquid chromatography tandem mass spectrometry (UPLC-MS/MS) method to quantify monotropein in blueberries

**DOI:** 10.1371/journal.pone.0329723

**Published:** 2025-11-21

**Authors:** Ishveen Kaur, Courtney P. Leisner, Dennis P. Cladis

**Affiliations:** 1 School of Plant and Environmental Sciences, Virginia Polytechnic Institute and State University, Blacksburg, Virginia United States of America; 2 Department of Food Science and Technology, Virginia Polytechnic Institute and State University, Blacksburg, Virginia United States of America; 3 Department of Human Nutrition, Foods, and Exercise, Virginia Polytechnic Institute and State University, Blacksburg, Virginia United States of America; Cairo University, EGYPT

## Abstract

Blueberries (*Vaccinium* species) are an economically important fruit crop rich in bioactive compounds like polyphenols and flavonoids. Interestingly, some blueberry cultivars also produce monotropein, which has bioactive properties, including anti-inflammatory and neuroprotective effects. However, methods to quantify monotropein in blueberries have not been optimized. To address this gap, an optimized analytical method for monotropein extraction and quantification using ultra performance liquid chromatography tandem mass spectrometry (UPLC-MS/MS) was developed. Different extraction strategies were compared, including variations in temperature, time, and ultrasonication treatments. Optimal extraction was achieved by heating samples to 60 °C for 15 mins in methanol. The method had high percent recovery and good repeatability. This protocol was then applied to 28 blueberry cultivars, 14 of which had not been previously analyzed for monotropein. Monotropein ranged from 0–1807 ng/mg dry weight. The developed method provides a robust tool that can be applied to future evaluations of monotropein in diverse blueberry cultivars.

## 1. Introduction

Plants produce two types of metabolites, broadly categorized into primary and secondary (specialized) metabolites. Primary metabolites are integral to plant growth, development, and reproduction [1]. These compounds are directly involved in energy production and contribute to plant growth and yield [2,3]. Plants also produce specialized metabolites that enable them to respond to environmental changes and can play key roles in defense against pests, pathogens, insects, and herbivores [4,5]. Most of these compounds are synthesized from intermediates of primary metabolic pathways. These plant specialized metabolites are broadly characterized into three main categories based on their function, structure, and biosynthesis: terpenes, (poly)phenols, and alkaloids [6].

Terpenes, characterized by the combination of one or more isoprene (C_5_H_8_) units, are the largest class of specialized metabolites, with over 30,000 unique compounds identified [7]. Iridoids are a subclass of terpenes that have the general form of a cyclopentanopyran [8]. Iridoids are widely distributed across various plant families and are typically found in the glycosylated form *in planta* [9,10]. Plants synthesize iridoids in response to biotic and abiotic stresses, protecting plants against herbivory, pathogen attacks, and environmental challenges such as temperature stress, extreme heat, temperature, UV, light, radiation [1]. When consumed by humans, iridoids may have health promoting properties, including anti-inflammatory, neuroprotective, antioxidant, antidiabetic, antimicrobial and cardioprotective activities [11,12].

Monotropein is an iridoid glycoside recognized as the principal bioactive compound in *Morinda officinalis* (family Rubiaceae), a plant widely used in traditional Chinese medicine (TCM) for the treatment of osteoporosis and inflammation [13–15]. Traditionally extracted from the roots of *M. officinalis*, monotropein has been reported to exhibit a broad spectrum of pharmacological activities, including antioxidant, anti-inflammatory, and anti-apoptotic effects [16,17]. Beyond TCM applications, monotropein is also employed in Polynesian ethnomedicine to manage a range of ailments such as infections, diabetes, asthma, hypertension, and pain [16]. These diverse therapeutic uses underscore the potential of monotropein as a multifunctional, medicinal, natural product.

Recent studies have identified the presence of monotropein in blueberries (*Vaccinium* species) [18–20]. Blueberries are an economically important fruit crop valued for their abundant phytochemical content and notable antioxidant activity [18,21–24]. In a comprehensive metabolite screen of an 84-member *Vaccinium* diversity panel, Leisner et al. [18] reported that monotropein was detectable in all 13 wild *Vaccinium* species analyzed but only 5 out of 71 cultivated blueberry accessions, suggesting that monotropein may be lost through cultivation of commercial blueberries [18].

While the Leisner et al. [18] study was the first to report the presence of monotropein in cultivated blueberries, the extraction protocol was not optimized. Therefore, the goal of the present study was to develop and validate a robust analytical protocol to quantify monotropein across different blueberry varieties. A systematic approach was used to optimize extraction efficiency, including the use of various temperatures, extraction durations, and the use of ultrasonication. After optimization, the protocol was applied to the quantification of monotropein in 28 blueberry accessions, many of which have never been analyzed for monotropein content.

## 2. Materials and methods

### 2.1. Materials and supplies

#### 2.1.1. Plant samples and materials.

All methods were developed using fruit from a lyophilized, monotropein-positive wild blueberry composite (WBC) supplied by the Wild Blueberry Association of America (WBANA). The optimized protocol was then applied to *Vaccinium* fruits (both wild and cultivated) obtained from the United States Department of Agriculture Germplasm Resources Information Network (USDA GRIN), in Corvallis, Oregon, USA. The samples collected from USDA GRIN represent *Vaccinium* spp. from several different countries of origin; accession identification information is shown in S1 Table. Samples were shipped from GRIN overnight on ice, flash frozen, and stored at −80 °C until analysis. A single biological replicate was obtained for all *Vaccinium* spp. from USDA GRIN. Additionally, samples of cultivated blueberry were also analyzed. This material was obtained from various public nurseries and breeding programs.

#### 2.1.2. Chemical materials and supplies.

A commercial standard of monotropein was purchased from Sigma-Aldrich (> 99% purity, CAS number 5945-50-6, St. Louis, MO, USA). LC-MS grade solvents, including methanol, acetonitrile, and formic acid were purchased from Thermo Fisher Scientific (Waltham, MA, USA). Type I ultrapure water (18.2 MΩ·cm) was used for all experiments (PureLab Flex, Elga Veolia, High Wycombe, United Kingdom).

### 2.2. Extraction optimization

For extraction optimization, 50 mg of WBC was mixed with 5 ml of LC-MS grade methanol and vortexed for 1 min. After vortexing, the samples were subjected to different treatments as described below and shown in Table 1. Samples were then centrifuged (Beckman Coulter Allegra V-15R, Brea, California, USA) for 5 mins at 1776x *g*; the supernatant was decanted and the remaining pellet extracted a second time. The supernatants were combined and dried completely under nitrogen using a nitrogen evaporator (Organomation N-Evap 116, Berlin, Massachusetts, USA). Dried extracts were resolubilized with 100 μL methanol and vortexed for 1 min. After vortexing, 1900 μL of ultrapure water was added before vortexing again, creating a 95:5 water:methanol mixture, which was matrix matched to the monotropein standard (see section 2.4.1). The resolubilized extract was filtered through 0.45 μm PTFE filters into LC vials and analyzed via UPLC-MS/MS, as described below.

**Table 1:** Extraction parameters tested.

Parameter	Variables tested
Time	15 min, 30 min, 45 min, 60 min
Temperature	Room Temperature, 30 °C, 45 °C, 60 °C
Heat x sonication	Heat alone, Sonication alone, Sonication followed by Heat, Heat followed by Sonication

Different parameters were used to optimize the extraction protocol for monotropein quantification. Heat, ultrasonication, and combination of both at different temperatures and time intervals were analyzed to optimize monotropein extraction (Table 1). For heating, a water bath (Fisherband- GPD05, Hampton, NH, USA) was used. Heating for different time intervals (15, 30, 45 and 60 mins) and at different temperatures (room temperature, 30, 45, and 60°C) to extract monotropein from the blueberry fruit tissue were evaluated. Additionally, an ultrasonication method was used to disrupt the cells using high frequency ultrasonic waves emitted by asonicator (Thermo Fisher Scientific, FS30D Ultrasonic Cleaner). Heat and ultrasonication methods were used independently as well as in different combinations to find the most effective method for a total of thirty-three treatment combinations for (see Table 1 for complete list).

### 2.3. UPLC-MS/MS analysis

Monotropein was quantified via Ultra-Performance Liquid Chromatography tandem Mass- spectrometry (UPLC-MS/MS) using a Waters UPLC Acquity H Class system equipped with a Xevo TQ-S micro detector (Waters Corporation, Milford MA, USA). Samples were injected and separated using an Acquity BEH C18 column (2.1 μm, 1.7 mm id x 50 mm) heated to 40°C and having a flow rate of 0.5 mL/min. Samples were eluted using a biphasic gradient of solvent A (0.1% formic acid inultrapure water) and solvent B (0.1% formic acid in acetonitrile) as follows: 0 min, 0% B; 1.0 min, 5% B; 1.5 min, 95% B; 2.5 min, 95% B; 2.6 min, 0% B; 4.0 min, 0% B. MS conditions were as follows: capillary voltage, 0.5 kV; source temp, 150 °C; desolvation temp, 600 °C; desolvation gas flow, 1000 L/h; cone gas flow, 50 L/h. Each of these parameters was optimized during method development, with the parameters that produced the strongest signal used to quantify monotropein. Identification and quantification of monotropein was based on the authentic standard, using calibration standards ranging from 0.001–100 µg/mL, though only standards above the LOQ_LCMS_ were included in the final calibration curve used to quantify monotropein. Monotropein (390.3 g/mol) had a retention time of 1.04 min and was detected using electrospray ionization operating in positive mode (ESI^+^) coupled with a triple quadrupole mass spectrometer. The parent ion (*m/z* 413.1) was detected as the sodium adduct of monotropein ([M + Na]^+^). A total of four mass transitions were optimized using IntelliStart software (413.117 → 185.020, 413.117 → 202.978, 413.117 → 233.036, and 413.117 → 251.056; S1–S2 Figs). The cone voltage for all transitions was 26 V, and the collision energies were 22, 26, 26, and 22 eV, respectively. The most abundant fragment (413.117 → 233.036) was used for quantification (S1–S2 Figs).

### 2.4. Method validation

The purpose of the method validation experiments described below was to establish a reliable and repeatable method for routine use in the author’s research laboratory. The method validation does not rise to the level of rigor necessary for use in a regulatory lab, as regulatory guidelines (e.g., IUPAC or SANTE/11312/2021) were not explicitly followed. However, the experimental approach was based on these guidelines and addresses the key elements stated in these guidelines, leading to the development and validation of a reliable and repeatable method that can be used in a research lab.

#### 2.4.1. Standard preparation and linearity.

The monotropein standard curve was prepared from pure monotropein powder (see section 2.1.2). Briefly, 10 mg monotropein was resolubilized using 1 mL methanol followed by 3 mL ultrapure water and quantitatively transferred to a volumetric flask where the dissolved monotropein was diluted with ultrapure water to a final concentration of 1000 μg/mL in 95:5 water:methanol (v/v). This standard was then serially diluted with 95:5 water:methanol to prepare a calibration curve (0.001 to 100 μg/mL) for UPLC-MS/MS that was matrix matched to the extracted and resolubilized sample (see section 2.2).

#### 2.4.2. Instrument detection limit (IDL), method detection limit (MDL), and limit of quantification (LOQ).

Limits of detection and quantification on the UPLC-MS/MS were estimated based on the standard deviation (σ) of five replicates each of five concentrations of the monotropein standard (0.004, 0.01, 0.04, 0.1, and 0.4 μg/mL), where the IDL was estimated as 3σ and LOQ_LCMS_ was estimated as 10σ, as previously described [25,26]. To back calculate the MDL and LOQ_blueberry_ to determine the detection and quantification limits in lyophilized blueberry fruits, the IDL and LOQ_LCMS_ were multiplied by the resolubilization volume (i.e., 2 mL) and divided by the mass of extracted blueberry tissue (i.e., 50 mg), as previously described [26].

#### 2.4.3. Repeatability.

Repeatability was determined as previously reported. Briefly, intraday repeatability was assessed by analyzing 3 concentrations of the monotropein standard (0.1, 1, and 10 μg/mL), 5 times on a single day. Interday repeatability was determined across 5 consecutive days by analyzing 3 concentrations of the monotropein standard (0.1, 1, and 10 μg/mL) 3 times per day. Repeatability was determined as the relative standard deviation (%RSD): (standard deviation/mean) x 100%, as previously described [26–28].

#### 2.4.4. Percent recovery.

Recovery was determined using three concentrations of the monotropein standard (0.1, 1, and 10 μg/mL) added to either blanks or WBC powder. Each was extracted in triplicate to determine recovery, as previously described [27,28]. After extraction and analysis, the % recovery was calculated by: (measured amount of monotropein standard)/(starting amount of monotropein) x 100%.

#### 2.4.5. Ion ratio.

The ion ratio was determined by calculating the ratio of peak area for the quantifying ion (413.117 → 233.036 *m/z*) to the qualifier ion (413.117 → 202.978 *m/z*). The ion ratio was estimated using peak areas from the experiment in 2.4.2. and a threshold of ± 15% applied to establish the acceptable range.

### 2.5. Statistical analysis

Data were analyzed for outliers, normality, homogeneity of variance and collinearity by analyzing homogeneity of variance, goodness of fit, and generating Q-Q plots. Due to the non-normal distribution of the data, the Kruskal–Wallis (KW) test was used. KW is a non-parametric alternative to one-way ANOVA and was used to evaluate differences between extraction strategies. The generalized linear model included time (15, 30, 45, and 60 min), treatment group (heat, sonication, heat then sonication, and sonication then heat), and temperature (room temperature, 30 °C, 45 °C, and 60 °C) as variables. All statistical analyses were performed in R version 4.3.1 [29]. To determine differences between monotropein content in wild versus cultivated varieties, the non-parametric Mann-Whitney test was used to due to the non-normal distribution of the data.

## 3. Results

### 3.1. Optimization of monotropein extraction conditions

To systematically optimize the extraction protocol, several techniques were used, including sonication, heat, and time (Table 1). These parameters were chosen based on previous extractions of specialized metabolites, including monotropein [18,30]. These extraction parameters were then systematically evaluated to determine the most efficient conditions. To start, heat alone, sonication alone, and the combination of both were compared. There were no significant differences in extraction efficiency (*p* = 0.19). Therefore, to streamline the method, the most time- and labor-intensive options (i.e., the sequential combinations of heat and sonication, regardless of their order) were eliminated.

Then, the effect of extraction time (i.e., 15, 30, 45, or 60 min) for the heat-only and sonication-only treatments were examined. Extraction time had no significant effect on extraction efficiency for either technique (*p* = 0.19). Thus, for both heat and sonication, only the 15-min treatment (i.e., the most efficient option that did not compromise extraction efficiency) was considered further. When evaluating the effect of temperature (i.e., 30 °C, 45 °C, or 60 °C) on extraction yield for the 15 min, heat-only treatment, the 60 °C treatment demonstrated significantly higher monotropein extraction (*p* = 0.008), making it the most favorable condition.

Finally, a direct comparison between the best heat-only condition (i.e., 60 °C for 15 min) and the best sonication-only condition (i.e., 15 min) showed that the heat-only condition was significantly better than the sonication-only condition (*p* = 0.041). Therefore, the heat-only condition at 60 °C for 15 mins was selected as the optimized method. Using the optimized method, the concentration of monotropein in WBC was determined to be 106 ng/mg.

### 3.2. Method validation

#### 3.2.1. Linearity, sensitivity, and limits of detection and quantification.

After optimizing the extraction method, the linearity and sensitivity of the UPLC-MS/MS method to detect monotropein were evaluated (Table 2). The linear range of monotropein was 0.04–40 μg/mL, with the calibration curve having a correlation coeffient of 0.9989. The instrument detection limit (IDL) is the smallest concentration of monotropein that can be detected on the UPLC-MS/MS, while the limit of quantification (LOQ_LCMS_) is the smallest concentration that can be quantified. Similarly, the method detection limit (MDL) is the smallest amount of monotropein that can be detected in the lyophilized blueberries themselves, while the limit of quantification (LOQ_blueberry_) is the smallest concentration that can be quantified. The IDL and LOQ_LCMS_ were 0.0103 and 0.0273 μg/mL, respectively, while the MDL and LOQ_blueberry_ were 0.412 and 1.092 μg/mL, respectively (Table 2). When factoring in the 1 μL injection volume used in this study, these limits are equivalent to IDL of 10.3 pg on column and LOQ_LCMS_ of 27.3 pg on column, both of which are similar to a previous report of IDL and LOQ for a triple quadropole instrument used to analyze iridoids [31] as well as other experiments measuring phytochemicals with a triple quadrupole mass spectrometer [32].

**Table 2:** Method validation parameters for the quantification of monotropein.

Parameter	Monotropein
Retention time	1.04 min
Linear range	0.04-40 μg/mL
Instrument detection limit (IDL)	0.0103 μg/mL
LOQ_LCMS_	0.0273 μg/mL
Method detection limit (MDL)	0.412 μg/g
LOQ_blueberry_	1.092 μg/g
Intraday recovery	89-110%
Intraday repeatability	1.17-2.15% RSD
Interday recovery	91-108%
Interday repeatability	4.68-7.16% RSD

Abbreviations: IDL = instrument detection limit; LOQ = limit of quantification; MDL = method detection limit; % RSD = percent relative standard deviation.

#### 3.2.2. Repeatability.

Intraday and interday repeatability of monotropein are shown in Table 2. Intraday %RSD was 1.17–2.15%, while interday %RSD was 4.68–7.16%.

#### 3.2.3. Recovery.

Recoveries of monotropein are shown in Table 2. Intraday recoveries were 89–110%, while interday recoveries were 91–108%.

#### 3.2.4. Ion ratio.

The ion ratio was 2.61 with an acceptable range of 2.22–3.00 using a tolerance of ± 15%. All standards with concentrations ≥ 10 ng/mL had ion ratios within the acceptable range. Additionally, all blueberry samples with quantifiable monotropein also had ion ratios within the acceptable range.

### 3.3. Monotropein quantification in 28 blueberry species

**Fig 1:**
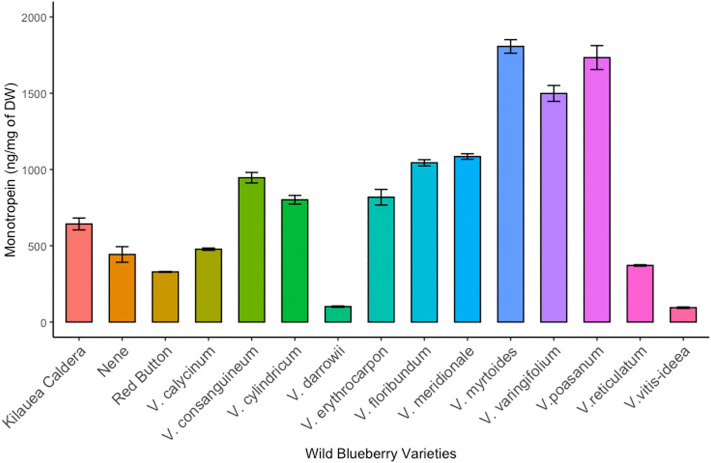
Monotropein content (µg/mg DW) in wild *Vaccinium* species. Error bars represent standard deviation (SD) from 3 technical replicates.

**Fig 2:**
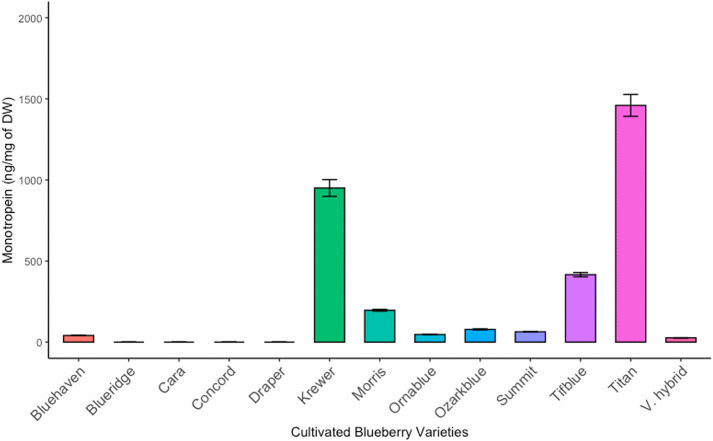
Monotropein content (µg/mg DW) in cultivated blueberries. Error bars represent standard deviation (SD) from 3 technical replicates.

The optimized method was applied to the quantification of monotropein in 28 blueberry fruit samples, including 15 wild and 13 cultivated varieties (Figs 1–2; Table 3; raw data in S2 Table). The range of monotropein content in wild *Vaccinium* species was 94–1807 ng monotropein/mg blueberry dry weight (dw), while in cultivated varieties monotropein ranged from below detection limits to 1460 ng/mg dw. Among the varieties analyzed here, wild *Vaccinium* species contained a significantly higher average concentration of monotropein than cultivated varieties (813 vs. 364 ng/mg dw; *p* = 0.0006), though several cultivated varieties contained more monotropein than some wild species.

**Table 3:** Monotropein quantification in blueberries.

Blueberry species	Ecotype	Monotropein content (ng/mg dw)	
		*This study*	*Leisner 2017* [18]
*Vaccinium calycinium*	Wild	477.5 ± 7.6	542.3
*V. reticulatum* Nene	Wild	442.9 ± 50.9	553.7
*V. reticulatum* Red Button	Wild	328.7 ± 3.3	256.6
*V. cylindricum*	Wild	801.6 ± 28.3	1387.4
*V. floribundum*	Wild	1044.0 ± 19.9	2040
*V. consanguineum*	Wild	946.1 ± 34.5	1707.4
*V. reticulatum* HIL-2009–005	Wild	371.4 ± 4.9	*n/a* ^*a*^
*V. erythrocarpon*	Wild	818.2 ± 51.1	*n/a*
*V.vitis-ideea*	Wild	94.1 ± 5.1	*n/a*
*V. varingifolium*	Wild	1498.6 ± 52.3	*n/a*
*V. myrtoides*	Wild	1806.7 ± 44.3	*n/a*
*V. meridionale*	Wild	1084.6 ± 18.3	*n/a*
*V. poasanum (Symphysia poasana)*	Wild	1733.5 ± 78.8	*n/a*
*V. darrowii*	Wild	101.0 ± 4.5	*n/a*
*V. reticulatum* Kilauea Caldera	Wild	642.9 ± 38.9	*n/a*
Concord	Cultivated – NH	ND ^*b*^	ND
Cara	Cultivated – NH	ND	ND
Ornablue	Cultivated – HH	47.0 ± 1.9	42
Ozarkblue	Cultivated – SH	78.4 ± 3.01	112
Bluehaven	Cultivated – NH	41.0 ± 0.4	46.7
Blueridge	Cultivated – SH	ND	41.7
Summit	Cultivated – RE	63.7 ± 1.9	200
*Vaccinium* hybrid	Cultivated	26.6 ± 0.7	*n/a*
Krewer	Cultivated – RE	950.2 ± 51.8	*n/a*
Titan	Cultivated – RE	1459.8 ± 67.6	*n/a*
Tifblue	Cultivated – RE	415.9 ± 13	*n/a*
Morris	Cultivated – SH	196.1 ± 5.5	*n/a*
Draper	Cultivated – NH	ND	*n/a*

^a^ n/a = not available; indicates sample was not available (and thus not measured) in Leisner 2017 [18] study.

^b^ ND = not detected; indicates sample was analyzed, but monotropein was below limit of detection.

Abbreviations: dw = dry weight; HH = half highbush; NH = Northern highbush; RE = Rabbiteye; SH = Southern highbush; V. = Vaccinium.

## 4. Discussion

In this experiment, the extraction and quantification of monotropein in lyophilized blueberry fruits was optimized and applied to the analysis of 28 blueberry cultivars (15 wild and 13 cultivated varieties). The most efficient extraction method was determined to be heating at 60°C for 15 mins. When evaluating longer extraction times and the potential use of sonication (with or without heat), there were no significant differences in extraction efficiency, though lower temperatures did decrease extraction efficiency. The final conditions are shorter than those previously reported [18] while also improving on the previous method to provide quantitative recovery of monotropein from blueberry fruits, as demonstrated by the high intra- and interday recovery values. In this study, targeted UPLC-MS/MS was employed to optimize the quantification of monotropein from blueberry species. The method appeared to have good sensitivity, with IDL 0.0103 µg/mL, MDL 0.412 µg/mL, LOQ_LCMS_ 0.0273 µg/mL, and LOQ_blueberry_ 1.092 μg/mL, resulting in the quantification of monotropein in 24 of the 28 samples evaluated.

To contextualize the results of the present study, they were compared to the results of monotropein in cultivated and wild blueberry fruits from Leisner et al. [18]. Both investigations showed that wild species had more monotropein, on average, than cultivated varieties. However, while the range of monotropein in wild *Vaccinium* species was similar between the two studies (94–1807 ng/mg dw (present study) vs 20–2371 ng/mg dw [18]), the results for cultivated species were different. The Leisner et al. investigation reported that monotropein in 66 of 71 cultivated varieties (i.e., 93%) was below the limit of detection, and of the 5 cultivated varieties that did contain quantifiable monotropein, the maximum observed value was 180. ng/mg dw [18]. There are very few other studies evaluating monotropein in *Vaccinium* species. Three recent reports identified monotropein conjugated with p-coumaric acid, though these three reports only identified these compounds in bilberry (*Vaccinium myrtillus L*.), but did not report on blueberries [33–35]. Jensen et al detected monotropein in several *Vaccinium* species of cranberry, bilberry, and blueberry, though it was not quantified [36]. Heffels et al. reported the values of monotropein in fresh tissue of *V. myrtillus* and *V. uliginosum* [19]. While these species were not measured in the present study, the range of values in Heffels et al. were less than those in the present study (0.8–4.4 mg/kg fresh weight) [19]. In the present study, monotropein was quantified on a dry weight basis, as the water content variability can limit direct comparisons among cultivars or species. Furthermore, measuring monotropein in fresh tissue could be problematic as enzyme activity in fresh tissue can lower metabolite levels prior to analysis. Other studies that have identified monotropein in *Vaccinium* species or identified the structure of monotropein did not quantify the abundance of monotropein directly [20,36].

In the present study, monotropein was below the limit of detection in only 4 of 13 cultivated varieties (i.e., 31%). In the 9 cultivars containing quantifiable monotropein, the average was 364 ng/mg dw, which is significantly higher than any cultivated variety in [18]. This is likely due to differences in the ecotypes analyzed in both studies, as the cultivars in [18] were primarily Northern Highbush (NH). As previously reported, cultivated varieties with wild *Vaccinium* parentage are more likely to contain monotropein [18]. Differences in the wild *Vaccinium* parentage in NH compared to southern highbush (SH) or rabbiteye (RE) ecotypes may explain why non-NH ecotypes are more likely to contain quantifiable levels of monotropein [18]. In the present study, the cultivated blueberries consisted of 4 NH and 9 non-NH ecotypes. Monotropein was only quantified in 1 of 4 NH cultivars, while it was quantified in 8 of 9 species belonging to other ecotypes, which aligns well with the hypothesis that the presence of monotropein in cultivated varieties is due to introgressions from wild species.

Interestingly, when comparing the 13 varieties (6 wild and 7 cultivated) that were analyzed in both [18] and the present investigation, monotropein levels were similar or slightly lower in the current samples (Table 3). The similarity across both studies provides additional support for the consistency of the method across multiple lab settings. Where differences were noted, there are likely due to environmental factors, including harvest year, soil composition, and both micro- and macro-environmental conditions, as prior studies have highlighted the role of environmental factors in modulating the production of specialized metabolites in plants [1,37–39]. Similar differences based on genetic and environmental factors in iridoid metabolites have been also observed in previous studies as well [19,20]. Other potential sources of variation include differences in seed source, sampling time, and interlab variations.

## 5. Conclusion and future directions

In this study, methods to extract and quantify monotropein in blueberries were successfully developed, validated, and applied to the analysis of 28 blueberry cultivars, 15 of which were analyzed for the first time. The results of the present study aligned well with those of a previous investigation of blueberry monotropein [18], supporting the observation that wild *Vaccinium* species contain higher levels of monotropein than cultivated varieties and that cultivated ecotypes with introgressions of wild *Vaccinium* species are more likely to contain monotropein.

Future studies focusing on elucidating the molecular mechanisms that produce monotropein, the influence of gene-environment (GxE) interactions on blueberry monotropein synthesis, and blueberry monotropein bioavailability in humans are needed. The regulatory mechanisms and metabolic pathways governing monotropein production in *Vaccinium* spp. has not been determined, which is key to exploring the biosynthetic potential to increase blueberry monotropein through targeted breeding or biotechnological approaches. Additionally, teasing out the relative contributions of genetic background, environmental factors (such as soil composition, climate variability, and cultivation practices), and their synergistic effects are key to understanding the observed variability in monotropein levels among cultivars and wild accessions. Finally, determining the *in vivo* metabolism and bioavailability of monotropein following blueberry consumption is needed to clarify the contribution of monotropein to the health-promoting properties of blueberries. Ultimately, integrating metabolomic profiling with genomics and breeding strategies will enable the development of cultivars with optimized monotropein content, which may enhance their functional and commercial value in the nutraceutical market.

## Supporting information

S1 Table Information of the accessions used in the study. (DOCX)

S2 Table Raw data for monotropein content in blueberry varieties tested in this study. (XLSX)

S1 Fig Sample multiple reaction monitoring (MRM) chromatograms for monotropein using an authentic standard. Four transitions were measured; 413.117 → 233.036 was used for quantification.
(PDF)

S2 Fig Sample multiple reaction monitoring (MRM) chromatograms of monotropein in wild blueberry composite (WBC). Values represent extraction using wild blueberry powder obtained from WBANA. A total of four mass transitions were optimized using IntelliStart software (413.117 → 185.020, 413.117 → 202.978, 413.117 → 233.036, and 413.117 → 251.056).
(PDF)

## References

[1] Singh S, Kaur I, Kariyat R. The multifunctional roles of polyphenols in plant-herbivore interactions. Int J Mol Sci. 2021;22(3):1442. doi: 10.3390/ijms22031442 33535511 PMC7867105

[2] Crozier A, Clifford MN, Ashihara H. Plant secondary metabolites: occurrence, structure and role in the human diet. Oxford: Blackwell Publishing; 2006.

[3] Zaynab M, Ahmad F, Khan A, Farooq S, Rahman M, Riaz M, et al. Role of primary metabolites in plant defense against pathogens. Microb Pathog. 2019;137:103743. doi: 10.1016/j.micpath.2019.103743 31499183

[4] Kumar K, Sharma M, Kaur R, Singh S. An overview of plant phenolics and their involvement in abiotic stress tolerance. Stresses. 2023;3(3):570–85. doi: 10.3390/stresses3030037

[5] Kaur I, Kariyat R. Trichomes mediate plant-herbivore interactions in two Cucurbitaceae species through pre- and post-ingestive ways. J Pest Sci (2004). 2023;96(3):1077–89. doi: 10.1007/s10340-023-01611-x 37168103 PMC10047472

[6] Uenoyama R, Miyazaki T, Hurst JL, Beynon RJ, Adachi M, Murooka T, et al. The characteristic response of domestic cats to plant iridoids allows them to gain chemical defense against mosquitoes. Sci Adv. 2021;7(4):eabd9135. doi: 10.1126/sciadv.abd9135 33523929 PMC7817105

[7] Davis EM, Croteau RB. Cyclization enzymes in the biosynthesis of monoterpenes, sesquiterpenes, and diterpenes. In: Leeper FJ, Vederas JC, editors. Biosynthesis: aromatic polyketides, isoprenoids, alkaloids. Berlin: Springer; 2000. pp. 53–95.

[8] Thabet AA, Ayoub IM, Youssef FS, Al-Sayed E, Efferth T, Singab ANB. Phytochemistry, structural diversity, biological activities and pharmacokinetics of iridoids isolated from various genera of the family Scrophulariaceae Juss. Phytomedicine Plus. 2022;2(3):100287. doi: 10.1016/j.phyplu.2022.100287

[9] Mabou F, Yossa I. Terpenes: structural classification and biological activities. IOSR J Pharm Biol Sci. 2021;16(3):25–40. doi: 10.9790/3008-1603032540

[10] Miettinen K, Dong L, Navrot N, Schneider T, Burlat V, Pollier J, et al. The seco-iridoid pathway from Catharanthus roseus. Nat Commun. 2014;5:3606. doi: 10.1038/ncomms4606 24710322 PMC3992524

[11] Srivastava A, Singh V. Biological action of essential oils (terpenes). Int J Biol Med Res. 2019;10(3):6854–9.

[12] Przybylska D, Kucharska AZ, Sozański T. A review on bioactive iridoids in edible fruits – from garden to food and pharmaceutical products. Food Rev Int. 2022;39(9):6447–77. doi: 10.1080/87559129.2022.2117375

[13] Choi J, Lee K-T, Choi M-Y, Nam J-H, Jung H-J, Park S-K, et al. Antinociceptive anti-inflammatory effect of monotropein isolated from the root of Morinda officinalis. Biol Pharm Bull. 2005;28(10):1915–8. doi: 10.1248/bpb.28.1915 16204945

[14] Wu M, Lai H, Peng W, Zhou X, Zhu L, Tu H, et al. Monotropein: a comprehensive review of biosynthesis, physicochemical properties, pharmacokinetics, and pharmacology. Front Pharmacol. 2023;14:1109940. doi: 10.3389/fphar.2023.1109940 36937894 PMC10017856

[15] Zhang ZG, Huang YF, Wang JW, Zhang YF, Zhang YL, Liu Y. Monotropein isolated from the roots of Morinda officinalis increases osteoblastic bone formation and prevents bone loss in ovariectomized mice. Fitoterapia. 2016;110:166–172. doi: 10.1016/j.fitote.2015.12.019 26996879

[16] Shi Y, Liu Z, Zhang Y, Jiang S, Wang J, Liang G. Monotropein attenuates oxidative stress via Akt/mTOR-mediated autophagy in osteoblast cells. Biomed Pharmacother. 2020;121:109583. doi: 10.1016/j.biopha.2019.109583 31698268

[17] Kim I-T, Park H-J, Nam J-H, Park Y-M, Won J-H, Choi J, et al. In-vitro and in-vivo anti-inflammatory and antinociceptive effects of the methanol extract of the roots of Morinda officinalis. J Pharm Pharmacol. 2005;57(5):607–15. doi: 10.1211/0022357055902 15901350

[18] Leisner CP, Rossi G, Woods F, Konkol J, Howe AC. Differential iridoid production as revealed by a diversity panel of 84 cultivated and wild blueberry species. PLoS One. 2017;12(6):e0179179. doi: 10.1371/journal.pone.0179179 PMC5469490 28609455

[19] Heffels P, Müller L, Schieber A, Weber F. Profiling of iridoid glycosides in Vaccinium species by UHPLC-MS. Food Res Int. 2017;100(Pt 3):462–8. doi: 10.1016/j.foodres.2016.11.018 28964369

[20] Ma C, Dastmalchi K, Flores G, Wu S-B, Pedraza-Peñalosa P, Long C, et al. Antioxidant and metabolite profiling of North American and neotropical blueberries using LC-TOF-MS and multivariate analyses. J Agric Food Chem. 2013;61(14):3548–59. doi: 10.1021/jf400515g 23547798

[21] Banerjee S, Nayik GA, Gull A. Blueberries. Antioxidants in fruits: properties and health benefits. Singapore: Springer; 2020. pp. 593–614.

[22] Ashique S, Mukherjee T, Mohanty S, Garg A, Mishra N, Kaushik M, et al. Blueberries in focus: exploring the phytochemical potentials and therapeutic applications. J Agric Food Res. 2024;18:101300. doi: 10.1016/j.jafr.2024.101300

[23] Rossi G, Woods FM, Leisner CP. Quantification of total phenolic, anthocyanin, and flavonoid content in a diverse panel of blueberry cultivars and ecotypes. horts. 2022;57(8):901–9. doi: 10.21273/hortsci16647-22

[24] Badjakov I, Ivanova V, Todorova M. Bioactive compounds in small fruits and their influence on human health. Biotechnol Biotechnol Equip. 2008;22(1):581–7. doi: 10.1080/13102818.2008.10817499

[25] IUPAC. IUPAC compendium of chemical terminology (the “Gold Book”). 3rd ed. International Union of Pure and Applied Chemistry; 2025 [cited 18 Oct 2025]. Available from: https://goldbook.iupac.org/

[26] Cladis DP, Hill Gallant KM, Kisch AR, Murphy KM. Elemental analysis of 346 varieties of quinoa: Development and validation of microwave plasma optical emission spectrometry (MP-OES) method. J Food Composit Anal. 2025;141:107270. doi: 10.1016/j.jfca.2025.107270

[27] Lipkie TE, Janasch A, Cooper BR, Hohman EE, Weaver CM, Ferruzzi MG. Quantification of vitamin D and 25-hydroxyvitamin D in soft tissues by liquid chromatography-tandem mass spectrometry. J Chromatogr B Analyt Technol Biomed Life Sci. 2013;932:6–11. doi: 10.1016/j.jchromb.2013.05.029 23811497 PMC3815525

[28] Lee EJ, Hong JK, Whang WK. Simultaneous determination of bioactive marker compounds from Gardeniae fructus by high performance liquid chromatography. Arch Pharm Res. 2014;37(8):992–1000. doi: 10.1007/s12272-013-0293-1 24277694

[29] R Core Team. R: A language and environment for statistical computing. Vienna (Austria): R Foundation for Statistical Computing; 2024.

[30] Furrer A, Cladis DP, Kurilich A, Manoharan R, Ferruzzi MG. Changes in phenolic content of commercial potato varieties through industrial processing and fresh preparation. Food Chem. 2017;218:47–55. doi: 10.1016/j.foodchem.2016.08.126 27719937

[31] Lockhart A, Simon JE, Wu Q. Stability study of Nepeta cataria iridoids analyzed by LC/MS. Phytochem Anal. 2024;35(7):1674–87. doi: 10.1002/pca.3410 39099156

[32] Yilmaz MA, Ertas A, Yener I, Akdeniz M, Cakir O, Altun M, et al. A comprehensive LC-MS/MS method validation for the quantitative investigation of 37 fingerprint phytochemicals in *Achillea* species: A detailed examination of *A. coarctata* and *A. monocephala*. J Pharm Biomed Anal. 2018;154:413–24. doi: 10.1016/j.jpba.2018.02.059 29602084

[33] Bujor O-C, Le Bourvellec C, Volf I, Popa VI, Dufour C. Seasonal variations of the phenolic constituents in bilberry (*Vaccinium myrtillus* L.) leaves, stems and fruits, and their antioxidant activity. Food Chem. 2016;213:58–68. doi: 10.1016/j.foodchem.2016.06.042 27451155

[34] Liu S, Marsol-Vall A, Laaksonen O, Kortesniemi M, Yang B. Characterization and quantification of nonanthocyanin phenolic compounds in white and blue bilberry (*Vaccinium myrtillus*) Juices and Wines Using UHPLC-DAD−ESI-QTOF-MS and UHPLC-DAD. J Agric Food Chem. 2020;68(29):7734–44. doi: 10.1021/acs.jafc.0c02842 32609509 PMC7497633

[35] Medic A, Smrke T, Hudina M, Veberic R, Zamljen T. HPLC-Mass spectrometry analysis of phenolics comparing traditional bilberry and blueberry liqueurs. Food Res Int. 2023;173:113373. doi: 10.1016/j.foodres.2023.113373 37803708

[36] Jensen HD, Krogfelt KA, Cornett C, Hansen SH, Christensen SB. Hydrophilic Carboxylic Acids and Iridoid Glycosides in the Juice of American and European Cranberries (*Vaccinium macrocarpon* and *V. oxycoccos*), Lingonberries (*V. vitis-idaea*), and Blueberries (*V. myrtillus*). J Agric Food Chem. 2002;50(23):6871–4. doi: 10.1021/jf0205110 12405790

[37] Docimo T, Celano R, Lambiase A, Di Sanzo R, Serio S, Santoro V, et al. Exploring influence of production area and harvest time on specialized metabolite content of Glycyrrhiza glabra leaves and evaluation of antioxidant and anti-aging properties. Antioxidants (Basel). 2024;13(1):93. doi: 10.3390/antiox13010093 38247517 PMC10812728

[38] Muthusamy M, Lee SI. Abiotic stress-induced secondary metabolite production in Brassica: opportunities and challenges. Front Plant Sci. 2024;14:1323085. doi: 10.3389/fpls.2023.1323085 38239210 PMC10794482

[39] Yang L, Wen K-S, Ruan X, Zhao Y-X, Wei F, Wang Q. Response of plant secondary metabolites to environmental factors. Molecules. 2018;23(4):762. doi: 10.3390/molecules23040762 29584636 PMC6017249

