# Supplementary material for: Development and validation of ultra performance liquid chromatography tandem mass spectrometry (UPLC-MS/MS) method to quantify monotropein in blueberries
Source: PLoS One. 2025 Nov 21;20(11):e0329723. doi: 10.1371/journal.pone.0329723 (PMC12637891; doi:10.1371/journal.pone.0329723)
Supplement: S1 Table — (DOCX) [file pone.0329723.s001.docx]

**Table S1.** Information of the accessions used in the study.

| **Accession** | **Inventory** | **Name** | **Taxon** | **Ecotype** | **Geography** |
| --- | --- | --- | --- | --- | --- |
| PI 657263 | CVAC 1814 .001 PL | *V. calycinium* Ha 2008-004 | *V. calycinum* | Wild | Hawaii, USA |
| PI 666683 | CVAC 1819 .001 PL | *V. reticulatum* Nene | *V. reticulatum* | Wild | Hawaii, USA |
| PI 666682 | CVAC 1818 .001 PL | *V. reticulatum* Red Button | *V. reticulatum* | Wild | Hawaii, USA |
| PI 618215 | CVAC 1368 .004_F PL | *V. cylindraceum* 6251097 | *V. cylindraceum* | Wild | Azores, Portugal |
| PI 554930 | CVAC 669 .001 PL | *V. floribundum* Ecuador | *V. floribundum* | Wild | Ecuador |
| PI 554736 | CVAC 588 .001 PL | *V. consanguineum* RBG Scotland 772948 | *V. consanguineum* | Wild | Costa Rica |
| PI 660965 | CVAC 1874 .001 PL | *V. reticulatum* HIL -2009-005 | *V. reticulatum* | Wild | Hawaii, USA |
| PI 688388 | CVAC 2333 .001 PL | *V. erythrocarpum VA-2018-046 MLBS* | *V. erythrocarpon* | Wild | Virginia, United States |
| PI 555384 | CVAC 790 .001 PL | *V. vitis-idaea* | *V. vitis-idaea* | Wild | Russian Federation |
| PI 666852 | CVAC 2020 .001 PL | *V. varingifolium* HS89-4 | *V. varingifolium* | Wild | Jawa, Indonesia |
| PI 666881 | CVAC 2059 .001 PL | *V. myrtoides* 1171092 | *V. myrtoides* | Wild | Philippines |
| PI 618123 | CVAC 1148 .001 PL | *V. meridonale* NC 3737 | *V. meridionale* | Wild | Colombia |
| PI 618191 | CVAC 1530 .001 PL | *V. poasanum* MZ7786 | *Symphysia poasana* | Wild | Alajuela, Costa Rica |
| PI 657193 | CVAC 1723 .001 PL | *V. darrowii* HL-2006-039 | *V. darrowii* | Wild | Florida, USA |
| PI 555197 | CVAC 780 .002 PL | *V.reticulatum* Kilauea Caldera | *V. kilauea caldera* | Wild | Hawaii, USA |
| N/A *^a^* | N/A | *Concord* | *V. corymbosum* | Cultivated - NH | Michigan, USA |
| N/A | N/A | *Cara* | *V. corymbosum* | Cultivated - NH | Michigan, USA |
| PI 554954 | CVAC 711 .001_F PL | Ornablue | *V.* hybrid | Cultivated - HH | West Virginia, USA |
| PI 666656 | CVAC 1321 .001_F PL | Ozarkblue | *V. corymbosum* | Cultivated - SH | Arkansas, USA |
| PI 554847 | CVAC 227 .001_F PL | Bluehaven | *V. corymbosum* | Cultivated - NH | Michigan, USA |
| PI 554869 | CVAC 314 .001_F PL | Blue Ridge | *V.* hybrid | Cultivated - SH | North Carolina, USA |
| PI 618181 | CVAC 1329 .001_F PL | Summit | *V.* hybrid | Cultivated – RE | Maryland, USA |
| PI 666675 | CVAC 1760 .001 PL | *V.*  hybrid | *V.* hybrid | Cultivated | Mississippi, USA |
| N/A | N/A | Krewer | *V. corymbosum* | Cultivated – RE | Auburn, USA |
| N/A | N/A | Titan | *V. ashei* | Cultivated – RE | Auburn, USA |
| PI 554698 | CVAC 233 .001_F PL | Tifblue | *V. virgatum* | Cultivated – RE | Georgia, USA |
| PI 618200 | CVAC 1352 .001_F PL | Morris | *V. ovatum* | Cultivated – SH | Oregon, USA |
| N/A | N/A | Draper | *V. corymbosum* | Cultivated - NH | Michigan, USA |

*Abbreviations: HH = half highbush; NH = Northern highbush; RE = Rabbiteye; SH = Southern highbush*

*^a^ N/A: lines obtained from growers of specific locations in USA*
