# Supplementary figures and images for: Development and validation of ultra performance liquid chromatography tandem mass spectrometry (UPLC-MS/MS) method to quantify monotropein in blueberries
Source: PLoS One. 2025 Nov 21;20(11):e0329723. doi: 10.1371/journal.pone.0329723 (PMC12637891; doi:10.1371/journal.pone.0329723)

10 ug/ml

XEVO-TQSmicro#QEG0995

28-Oct-2024

15:47:12

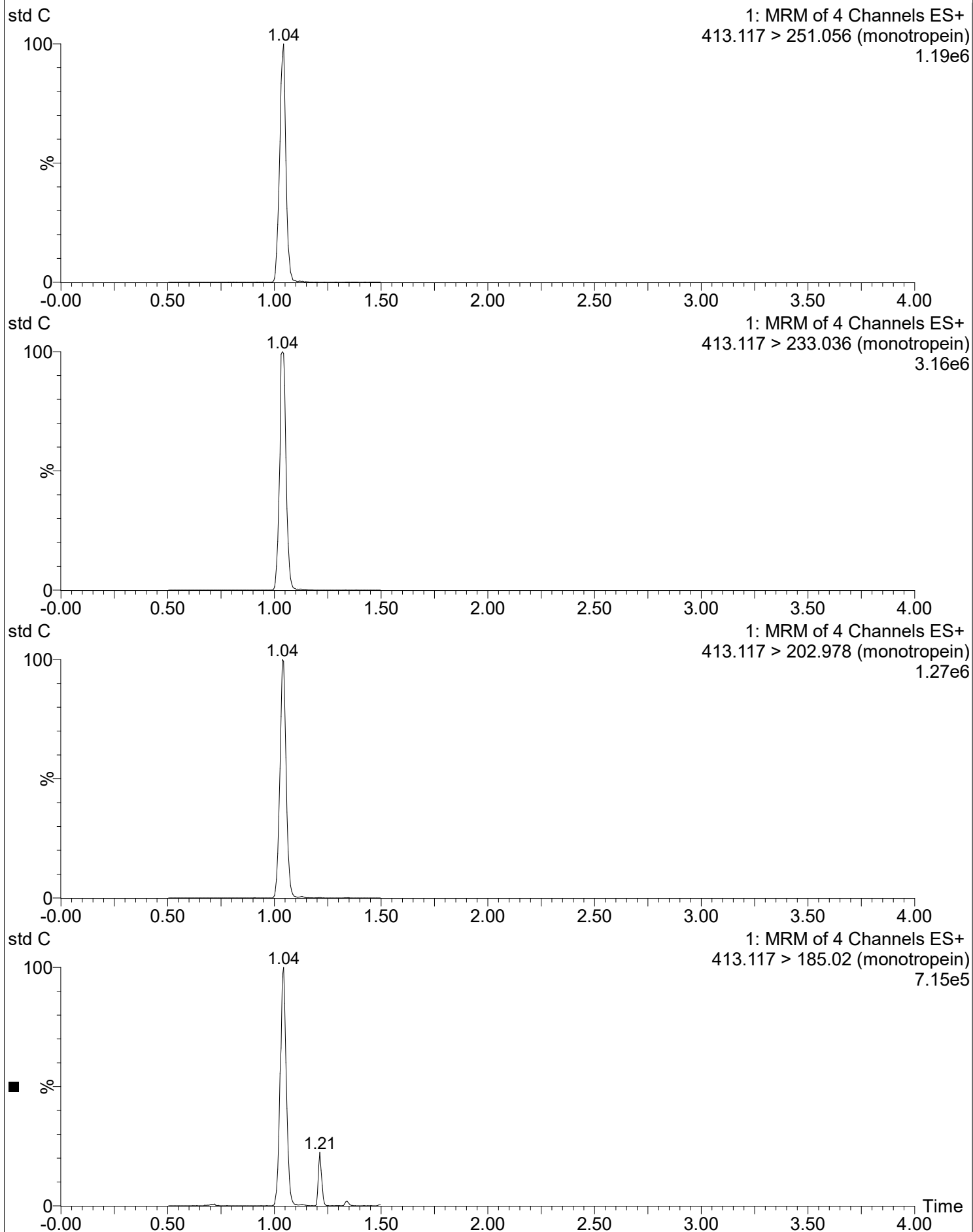

Supplement: S1 Fig — Four transitions were measured; 413.117 → 233.036 was used for quantification. (PDF) [file pone.0329723.s003.pdf]
