# Supplementary material for: Development and validation of ultra performance liquid chromatography tandem mass spectrometry (UPLC-MS/MS) method to quantify monotropein in blueberries
Source: PLoS One. 2025 Nov 21;20(11):e0329723. doi: 10.1371/journal.pone.0329723 (PMC12637891; doi:10.1371/journal.pone.0329723)

XEVO-TQSmicro#QEG0995 **25-Jul-2024**
12:47:14

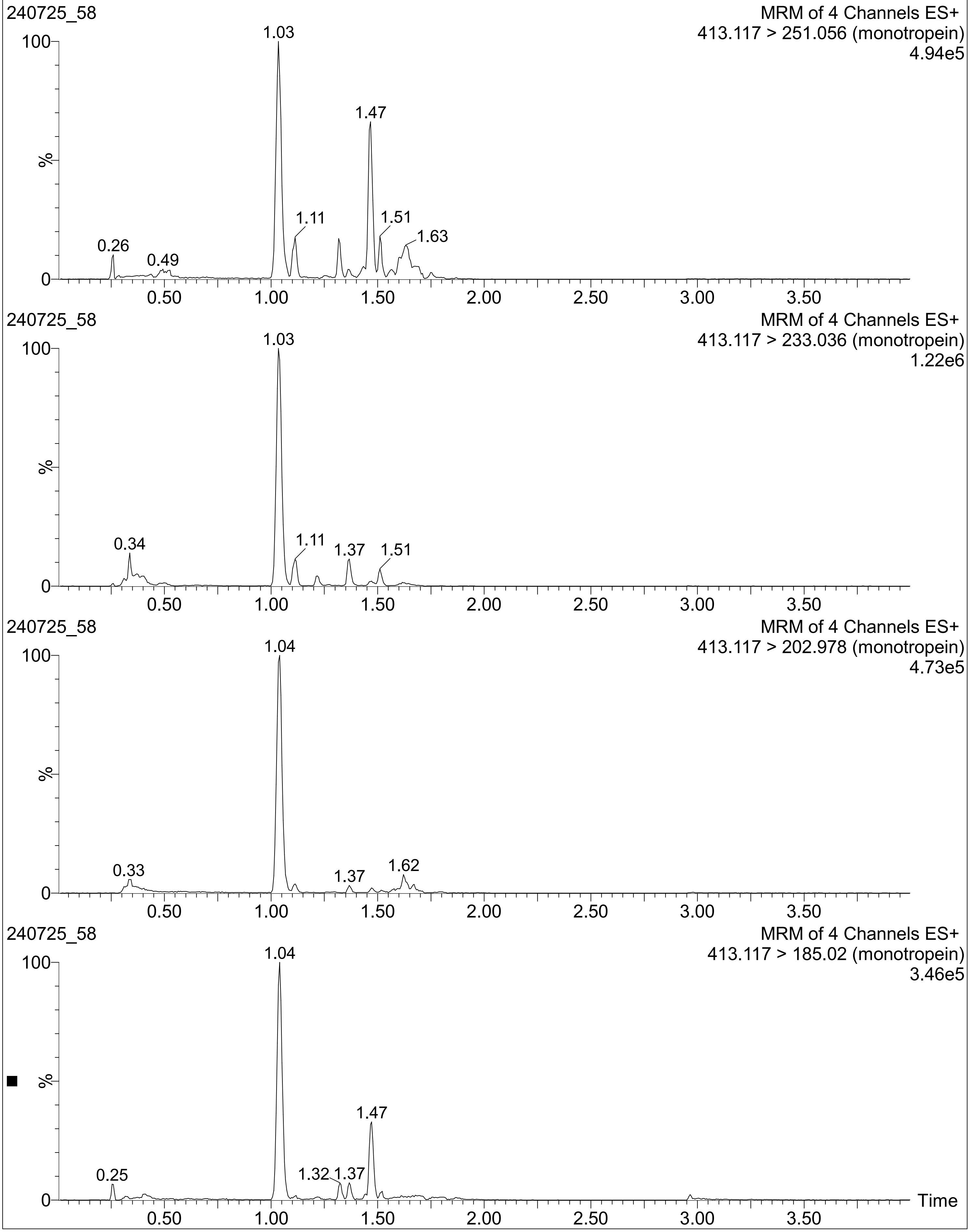

Supplement: S2 Fig — Values represent extraction using wild blueberry powder obtained from WBANA. A total of four mass transitions were optimized using IntelliStart software (413.117 → 185.020, 413.117 → 202.978, 413.117 → 233.036, and 413.117 → 251.056). (PDF) [file pone.0329723.s004.pdf]
